# Supplementary material for: Content-rich biological network constructed by mining PubMed abstracts
Source: BMC Bioinformatics. 2004 Oct 8;5:147. doi: 10.1186/1471-2105-5-147 (PMC528731; doi:10.1186/1471-2105-5-147)
Supplement: Additional File 2 — The original results of the above study (non-essential files are deleted to keep the file size under the limit set by BMC bioinformatics). [file 1471-2105-5-147-S2.bz2 › chilibotAdditionalFile2/dip20/504ID10880444E10671/html/BMP2_BMPR2.html]

 


 **BMP2** and **BMPR2** 
  
Found 51 abstracts in PubMed, retrieved 20.  
 

 What does Google say? 
 PDF only 
| .edu only 

---

**Interactive relationship** (e.g. stimulation, inhibition, etc)

**Neutral relationship**- Requirement of BMP 2  [ **BMP2** ]  induced phosphatidylinositol 3 kinase and Akt serine threonine kinase  [ **BMPR2** ]  in osteoblast differentiation and Smad dependent BMP 2  [ **BMP2** ]  gene transcription.  Ref: 12084724 J Biol Chem, 2002
- The binding epitopes of BMP 2  [ **BMP2** ]  for BMPR IA type I and BMPR II  [ **BMPR2** ]  or ActR II type II were characterized using BMP 2  [ **BMP2** ]  mutant proteins for analysis of interactions with receptor ectodomains.  Ref: 10880444 EMBO J, 2000
- BMP 2  [ **BMP2** ]  antagonists emerge from alterations in the low affinity binding epitope for receptor BMPR II  [ **BMPR2** ] .  Ref: 10880444 EMBO J, 2000
- METHODS To identify amino acid side chains involved in receptor binding, a collection of in vitro mutagenized human BMP 2  [ **BMP2** ]  variants was prepared and subjected to interaction analyses with use of the receptor ectodomains of BMPR IA, BMPR II  [ **BMPR2** ] , and ActR II immobilized on a biosensor system.  Ref: 11263668 J Bone Joint Surg Am, 2001
- BMP 2  [ **BMP2** ]  stimulated Akt serine threonine kinase  [ **BMPR2** ]  activity in a PI 3 kinase dependent manner in osteoblast precursor cells.  Ref: 12084724 J Biol Chem, 2002

**Non-interactive relationship** (e.g. studied together, co-existance, homology, etc.)

- **BMP2**  and BMP4 are produced by the thymic stroma and the requisite BMP receptor molecules BMPR 1A, BMPR 1B, BMPR II  [ **BMPR2** ] , and signal transduction molecules Smad, 8, and 4 are expressed by DN thymocytes.  Ref: 12421925 J Immunol, 2002
- With use of immunohistochemical analysis, we studied the localization of BMP 2  [ **BMP2** ] , BMP 4, and BMP 7 and their receptors BMPR IA, BMPR IB, and BMPR II  [ **BMPR2** ]  as well as pSmad1.  Ref: 12429748 J Bone Joint Surg Am, 2002
- Addition of the BMPR II  [ **BMPR2** ]  specific ligands,  **BMP2**  and BMP4, in the culture medium of the endothelial cells seeded on type I collagen increased 3 H thymidine incorporation into cellular DNA, indicating that endothelial cells were able to form a functional receptor.  Ref: 11327700 Biochem Biophys Res Commun, 2001
